# Link between capacity for current production and syntrophic growth in *Geobacter* species

**DOI:** 10.3389/fmicb.2015.00744

**Published:** 2015-07-21

**Authors:** Amelia-Elena Rotaru, Trevor L. Woodard, Kelly P. Nevin, Derek R. Lovley

**Affiliations:** ^1^Department of Microbiology, University of MassachusettsAmherst, MA, USA; ^2^Nordic Center for Earth Evolution, Department of Biology, University of Southern DenmarkOdense, Denmark

**Keywords:** *Geobacter*, *Methanosarcina*, syntrophy, direct interspecies electron transfer, electrogen

## Abstract

Electrodes are unnatural electron acceptors, and it is yet unknown how some *Geobacter* species evolved to use electrodes as terminal electron acceptors. Analysis of different *Geobacter* species revealed that they varied in their capacity for current production. *Geobacter metallireducens* and *G. hydrogenophilus* generated high current densities (ca. 0.2 mA/cm^2^), comparable to *G. sulfurreducens*. *G. bremensis*, *G. chapellei*, *G. humireducens*, and *G. uraniireducens*, produced much lower currents (ca. 0.05 mA/cm^2^) and *G. bemidjiensis* was previously found to not produce current. There was no correspondence between the effectiveness of current generation and Fe(III) oxide reduction rates. Some high-current-density strains (*G. metallireducens* and *G. hydrogenophilus*) reduced Fe(III)-oxides as fast as some low-current-density strains (*G. bremensis*, *G. humireducens*, and *G. uraniireducens*) whereas other low-current-density strains (*G. bemidjiensis* and *G. chapellei*) reduced Fe(III) oxide as slowly as *G. sulfurreducens*, a high-current-density strain. However, there was a correspondence between the ability to produce higher currents and the ability to grow syntrophically. *G. hydrogenophilus* was found to grow in co-culture with *Methanosarcina barkeri*, which is capable of direct interspecies electron transfer (DIET), but not with *Methanospirillum hungatei* capable only of H_2_ or formate transfer. Conductive granular activated carbon (GAC) stimulated metabolism of the *G. hydrogenophilus – M. barkeri* co-culture, consistent with electron exchange via DIET. These findings, coupled with the previous finding that *G. metallireducens* and *G. sulfurreducens* are also capable of DIET, suggest that evolution to optimize DIET has fortuitously conferred the capability for high-density current production to some *Geobacter* species.

## Introduction

*Geobacter* species are among the most effective microorganisms for harvesting electrical current from organic compounds (Call and Logan, 2011; Lovley et al., 2011; Kumar et al., 2015). However, the electrodes that serve as the electron acceptors in current production are not found in the soils and sediments that are the natural habitat of *Geobacter* species. Therefore, the selective pressure to optimize the reduction of other extracellular electron acceptors, which *Geobacter* species naturally utilize, may have fortuitously lead to the superior ability of *Geobacter* species to produce high current densities. If the natural analog for electrodes could be identified this could aid in understanding of the mechanisms for electron transfer to electrodes as well as guide strategies to improve the current production capabilities of *Geobacter* species. Two potential natural analogs are poorly crystalline insoluble Fe(III) oxides and direct interspecies electron transfer (DIET) partners.

The primary electron acceptor for *Geobacter* species in many soils and sediments is poorly crystalline insoluble Fe(III) oxides (Lovley et al., 2011). Electrodes and Fe(III) oxides are both extracellular electron acceptors and therefore it is possible that the evolution of *Geobacter* species to excel at Fe(III) oxide reduction also yielded characteristics for effective current production. However, there are important differences in the properties of electrodes and Fe(III) oxides. For example, a current-harvesting electrode provides a long-term, stable electron sink for *Geobacter* respiration whereas an Fe(III) oxide particle has a limited, finite capacity to accept electrons. Furthermore, electrodes are typically much larger than *Geobacter* cells, whereas most Fe(III) oxide minerals in soils, as well as the poorly crystalline Fe(III) oxides typically employed in culture medium (Lovley and Phillips, 1988), are much smaller than the cells. Therefore, *Geobacter* species cannot form biofilms on Fe(III) oxides and motile cells that can search for new Fe(III) oxide sources appear to have a selective advantage in Fe(III) oxide reduction (Childers et al., 2002; Tremblay et al., 2012; Ueki et al., 2012). This is evident in subsurface environments in which *Geobacter* species are actively reducing Fe(III) oxides, where the cells are readily recovered in the groundwater (Anderson et al., 2003; Holmes et al., 2007, 2015). In contrast, *Geobacter* species oxidizing organic compounds with an electrode as the electron acceptor attach to the electrode surface and can form biofilms many cell layers thick (Reguera et al., 2006; Nevin et al., 2008; Richter et al., 2008; Franks et al., 2012).

A more appropriate natural analog for *Geobacter* electrode biofilms might be the cell aggregations established during DIET. When *Geobacter metallireducens* and *G. sulfurreducens* were grown in co-culture in a medium, which contained an electron donor that only *G. metallireducens* could metabolize (ethanol) and an electron acceptor that only *G. sulfurreducens* could reduce (fumarate), the two species formed large (1 mm diameter) aggregates (Summers et al., 2010; Shrestha et al., 2013a). The aggregates were electrically conductive (Summers et al., 2010), similar to anode biofilms (Malvankar et al., 2011). Conductive *Geobacter*-rich aggregates have been noted in anaerobic digesters converting organic wastes to methane (Morita et al., 2011; Shrestha et al., 2014) and defined co-cultures of *G. metallireducens* and either *Methanosaeta* (Rotaru et al., 2014b) or *Methanosarcina* (Rotaru et al., 2014a) species form visible aggregates to share electrons via DIET. The abundance of *Geobacter* species in some methanogenic soils and sediments (Hori et al., 2007; Kato et al., 2012; Xu et al., 2013) as well as the ability of conductive minerals to simultaneously enhance the growth of *Geobacter* species and methane production (Kato et al., 2012; Liu et al., 2012; Chen et al., 2014a,b; Cruz Viggi et al., 2014; Li et al., 2014; Rotaru et al., 2014a; Shrestha and Rotaru, 2014) suggests that co-aggregation of *Geobacter* species and methanogens may be a common phenomenon in these methanogenic environments as well.

Although the details of long-range electron transfer through current-producing biofilms and aggregates involved in DIET are still being elucidated, the electrically conductive pili of *Geobacter* species, known as microbial nanowires, are central to both processes as well as for Fe(III) oxide reduction (Reguera et al., 2005, 2006; Nevin et al., 2009; Tremblay et al., 2012; Vargas et al., 2013; Lovley and Malvankar, 2015). Studies of *G. sulfurreducens* pili have revealed that pili possess metallic-like conductivity (Malvankar et al., 2011), which can be attributed to overlapping π–π orbitals of aromatic amino acids (Vargas et al., 2013; Lovley and Malvankar, 2015). Genetically eliminating the capacity for pili production (Reguera et al., 2005; Tremblay et al., 2012) or diminishing pili conductivity (Vargas et al., 2013; Liu et al., 2014) severely reduces Fe(III) oxide reduction and current production, whereas increasing expression of pili yields higher currents (Yi et al., 2009; Leang et al., 2013). In a similar manner, co-culture aggregates sharing electrons via DIET could not be established with a strain of *G. metallireducens* that could not produce pili (Shrestha et al., 2013b; Rotaru et al., 2014a,b).

Other outer-surface proteins, including *c*-type cytochromes, are also required for extracellular electron transfer to Fe(III) oxides, electrodes, or syntrophic partners (Lovley et al., 2011). However, the lack of a full understanding of how all these components interact, especially in biofilms and aggregates, has made it as yet impossible to make direct comparison of the mechanisms for electron transfer to Fe(III) oxides, other cells, and electrodes.

Previous studies on current production, Fe(III) oxide reduction, and DIET by *Geobacter* species have primarily focused on *G. sulfurreducens*, because it is closely related to the *Geobacter* species that often predominate in current-harvesting biofilms and because it can readily be genetically manipulated (Lovley et al., 2011; Mahadevan et al., 2011). Therefore, in order to gain insight into whether Fe(III) oxide reduction or DIET might be a better natural analog for electron transfer to electrodes, we compared the ability of a diversity of other *Geobacter* species to produce current, reduce Fe(III) oxide, and participate in DIET.

## Materials and Methods

### Source of Organisms and Routine Cultivation

All *Geobacter* species were from our laboratory culture collection. *Methanosarcina barkeri* (DSM 800) was purchased from the German Culture Collection.

Cultivation was performed using strict anaerobic cultivation protocols (Balch et al., 1979). With the exception of cultivation with Fe(III) oxide as the electron acceptor (see below), all media were boiled and then cooled under N_2_:CO_2_ (80:20) to remove dissolved oxygen. The medium was dispensed in culture tubes and sterilized under a N_2_:CO_2_ (80:20) atmosphere. Substrates and vitamins were added from anaerobic, filtered sterilized stocks after the medium was autoclaved.

For routine cultivation *G. metallireducens*, *G. humireducens*, *G. hydrogenophilus*, *G. bremensis*, *G. bemidjiensis*, and *G. sulfurreducens* were provided with 50 mM Fe(III) citrate as electron acceptor, as previously described (Lovley and Phillips, 1988; Coates et al., 2001; Straub and Buchholz-Cleven, 2001; Nevin et al., 2005; Tremblay et al., 2012), with the exception that Fe(III) citrate was added from an anaerobic, sterile stock after the medium was sterilized. *G. uraniireducens* (Shelobolina et al., 2008), and *G. chapellei* (Coates et al., 2001), which can not use Fe(III) citrate as an electron acceptor, were provided with 40 mM fumarate as electron acceptor.

*Methanosarcina barkeri* was cultured anaerobically with 30 or 40 mM acetate as substrate on a modified DSMZ medium 120, as previously described (Rotaru et al., 2014a).

### Current Production

The capacity for current production was determined in flow-through, two-chambered H-cell systems with graphite stick anodes (65 cm^2^) poised at 300 mV, with a continuous supply of fresh acetate (10 mM) medium, as previously described (Nevin et al., 2009). Briefly, *Geobacter* species other than *G. metallireducens*, were pre-grown in fumarate (40 mM) and acetate (10 mM) media in the anode chamber and then the medium was replaced with medium containing only acetate (10 mM). For *G. metallireducens*, which does not grow on fumarate, cells were pre-grown in media containing Fe(III) citrate (55 mM) and acetate (10 mM), harvested by centrifugation, resuspended in bicarbonate buffer (30 mM), and inoculated into the anode chamber containing acetate (10 mM) medium.

### Fe(III) Oxide Reduction

All *Geobacter* species were adapted to grow effectively on ethanol or lactate prior to Fe(III)-oxide reduction tests for at least three transfers. All cultures grew overnight on these substrates if the electron acceptor was Fe(III)-citrate. Cultures were grown with poorly crystalline Fe(III) oxide (100 mmol/liter) as the electron acceptor as previously described (Lovley and Phillips, 1988) with the exception that ethanol (20 mM) was the electron donor for all cultures expect *G. sulfurreducens* which is unable to utilize ethanol and was provided with lactate (10 mM) as the electron donor.

### Co-Cultivation with *M. barkeri*

Prior to growth in co-cultures the *Geobacter* species were adapted to grow using ethanol for at least three transfers. We choose ethanol because it is the only known DIET-syntrophic substrate (Rotaru et al., 2014a,b).

Co-cultures of *Geobacter* species and *M. barkeri* were initiated in medium with ethanol (20 mM) as the electron donor and carbon dioxide as the only potential electron acceptor, as previously described (Rotaru et al., 2014a). Co-cultures were initiated with a 5% inoculum of each partner organism grown to mid- or late-logarithmic, as previously described (Rotaru et al., 2014a).

In order to evaluate the impact of granular activated carbon (GAC) on co-culture growth 0.1 g of GAC was added to the culture tubes along with 0.2 ml ultrapure water, sealed and sterilized at 121°C, under a N_2_:CO_2_ atmosphere for 1 h. Then 9 ml of medium and 5% inoculum of each partner organism were added to the anoxic, sterile tubes.

### Analytical Measurements

Samples for metabolite analyses were retrieved with hypodermic needles and syringes flushed with N_2_-CO_2_. For methane analysis, headspace samples (0.5 ml) were retrieved with a gas tight syringe and injected immediately on a Shimadzu gas chromatograph as previously described (Rotaru et al., 2014a). For ethanol and short chain volatile fatty acid analysis, 0.2 ml culture of medium was sampled aseptically and anaerobically with sterile pre-flushed syringes. Ethanol was measured on a gas chromatograph equipped with a flame ionization detector as previously described (Rotaru et al., 2014a). Short chain fatty acids were quantified with high performance liquid chromatography using a fast acid column (Rotaru et al., 2014a).

In cultures grown with Fe(III) oxide as terminal electron acceptor, Fe(II) was monitored with the ferrozine assay as previously described (Lovley and Phillips, 1987; Anderson and Lovley, 1999).

## Results and Discussions

### Current-Producing Capacity of Diverse *Geobacter* Species

**FIGURE 1:**
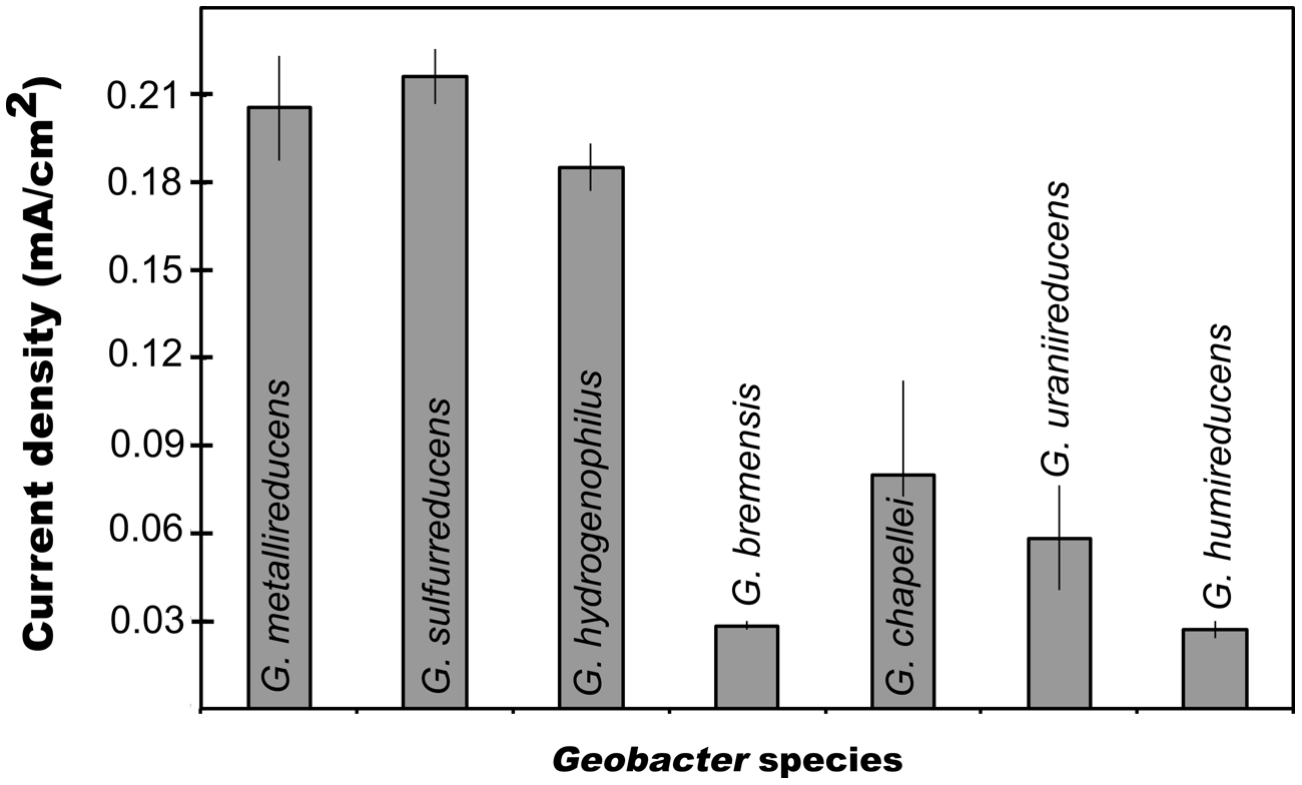
**Maximum current production for different *Geobacter* species.** Results are means of duplicate determinations.

In order to evaluate possible links between the capacity for current production and either Fe(III) oxide reduction or DIET, each of these processes were studied in seven species other than *G. sulfurreducens*. *G. metallireducens* (**Figure 1**) and *G. hydrogenophilus* (**Figure 1**) both produced currents (ca. 0.2 mA/cm^2^) comparable to those previously reported for *G. sulfurreducens* (Nevin et al., 2009). However, all the other strains tested generated much lower (ca. 0.05 mA/cm^2^) maximum currents (**Figure 1**). Furthermore, *G. bemidjiensis* was unable to produce current (Nevin et al., 2005). These results demonstrate that not all *Geobacter* species are highly effective current producers. The best current producers were *G. sulfurreducens*, *G. metallireducens*, and *G. hydrogenophilus*, which are closely related (Lovley et al., 2011). This suggests that common physiological factors specific to the evolution of these species confer the capacity for exceptional current production. These results may also explain why many studies have found that *Geobacter* species closely related to *G. sulfurreducens* predominate on electrodes harvesting electricity from mixed microbial communities (Lovley et al., 2011; Yates et al., 2012).

### Fe(III) Oxide Reduction Capabilities

**FIGURE 2:**
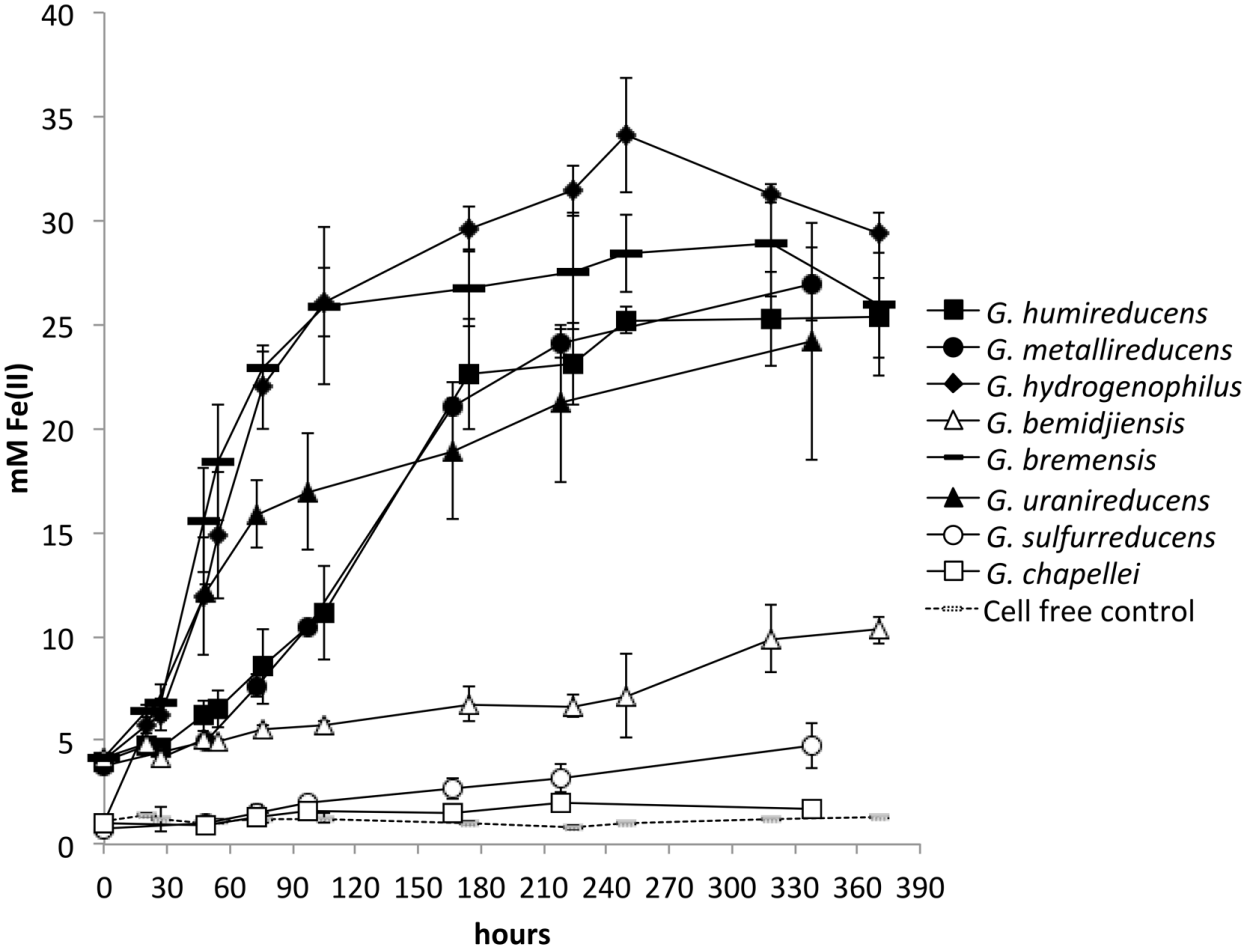
**Fe(II) production from insoluble Fe(III) oxides by different *Geobacter* species.** All species except for *Geobacter sulfurreduc*ens were provided with ethanol as electron donor. *G. sulfurreduc*ens cannot grow on ethanol, and was provided with 10 mM lactate as electron donor. Results are the mean and SD of triplicate cultures for each species.

In order to determine whether there was any correspondence between the effectiveness of current production and the ability to reduce insoluble Fe(III) oxides, each of the *Geobacter* species was grown in medium with insoluble Fe(III) oxide as the sole electron acceptor. The inoculum for each *Geobacter* strain grew rapidly overnight in their medium with soluble electron acceptor, but there were marked differences in the rate of metabolism in Fe(III) oxide medium. *G. chapellei* and *G. bemidjiensis*, two species that produced low currents, slowly reduced Fe(III) oxide slowly with maximum rates of Fe(II) production of 0.02 ± 0.06 and 0.04 ± 0.07 mM Fe(II) per hour, respectively (**Figure 2**). However, three other species with low current outputs, *G. bremensis*, *G. humireducens*, and *G. uraniireducens*, were highly effective Fe(III) oxide reducers with maximum Fe(III) oxide reduction rates of 0.44 ± 0.12, 0.16 ± 0.05, and 0.23 ± 0.03 mM Fe(II) per hour, respectively (**Figure 2**). *G. metallireducens* and *G. hydrogenophilus*, which produced high current densities were also very proficient insoluble Fe-oxide reducers (**Figure 2**) with maximum Fe(III) oxide reduction rates of 0.15 ± 0.02, and 0.33 ± 0.16 mM Fe(II) per hour, respectively (**Figure 2**). In contrast *G. sulfurreducens*, which is also highly effective in current production (Nevin et al., 2009), slowly reduced Fe(III) oxide [0.02 ± 0.06 mM Fe(II) per hour]. These results demonstrated that there is no correspondence between the capacities for Fe(III) oxide reduction and current production among these eight *Geobacter* species.

The reason for the between species differences in rates of Fe(III) oxide reduction require further investigation, but may be related to distinct selective pressures in the diverse environments from which these *Geobacter* species have been isolated. Furthermore, the different enrichment and isolation procedures by which many of these pure cultures were obtained may have selected for unique physiological characteristics which are reflected in the range of Fe(III) oxide reduction rates observed. One indication of this possibility is the lack of between species conservation in the *c*-type cytochromes likely to be involved in extracellular electron transfer (Butler et al., 2010).

### Syntrophic Growth with *M. barkeri*

Previous studies have demonstrated that both *G. metallireducens* and *G. sulfurreducens*, which produce high current densities, are also capable of DIET. *G. sulfurreducens* directly accepted electrons from *G. metallireducens* (Summers et al., 2010). *G. metallireducens* was capable of serving as the electron-donating partner in DIET with either *G. sulfurreducens* (Summers et al., 2010), *Methanosaeta harundinacea* (Rotaru et al., 2014b) or *M. barkeri* (Rotaru et al., 2014a) as the electron-accepting partner.

**FIGURE 3:**
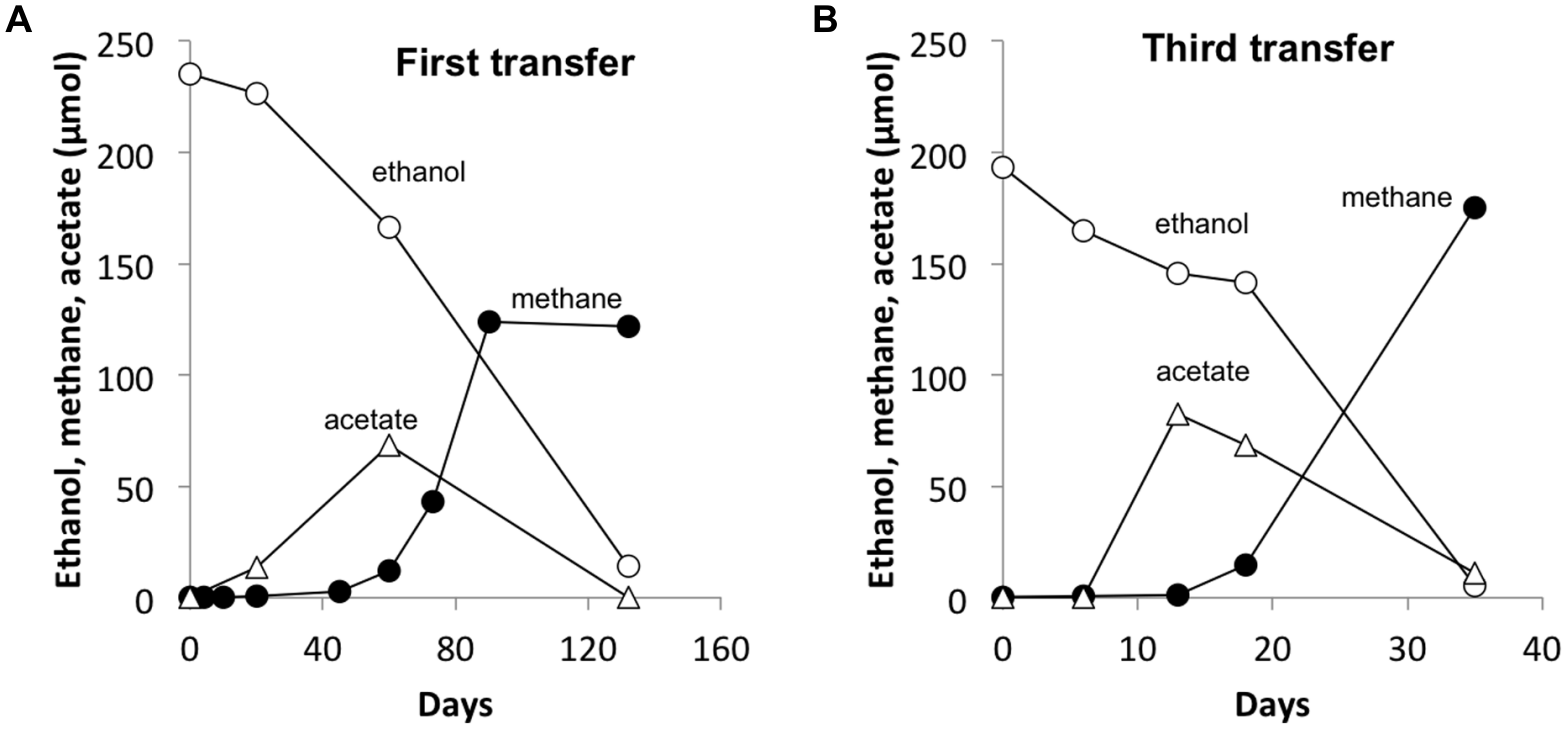
**A representative co-culture of *G. hydrogenophilus – Methanosarcina barkeri* during the first **(A)** and the third transfer **(B)**.** Notice the four-time decrease in time scale from the first to the third transfer. Values are from single incubations. See Supplementary Materials for all three replicate incubations.

**FIGURE 4:**
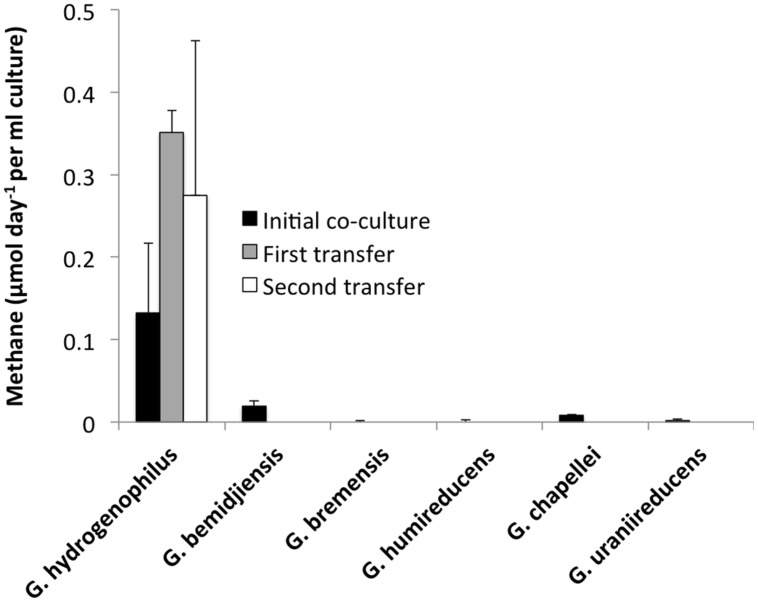
**Rates of methane evolution from ethanol in co-cultures of *M. barkeri* with different *Geobacter* species during the initial co-cultivation (black bars) and the subsequent two transfers for the successful *G. hydrogenophilus – M. barkeri* co-culture.** Results show the means and SD for triplicate co-cultures of each *Geobacter* species, incubated for a minimum of 100 days.

In order to determine if any other *Geobacter* species might function in a similar manner, co-cultures were initiated with *M. barkeri*. Of the *Geobacter* species evaluated, only *G. hydrogenophilus* successfully established co-cultures with *M. barkeri* (**Figures 3** and **4**), whereas G. *bemidjiensis*, *G. bremensis*, *G. chapellei*, *G. humireducens*, and *G. uraniireducens* did not (**Figure 4**).

As previously observed with co-cultures established between *G. metallireducens* and *M. barkeri* (Rotaru et al., 2014b), there was a long lag prior to detectable methane production *in G. hydrogenophilus–M. barkeri* co-cultures (**Figure 3A**). However, over time the co-culture adapted to steadily produce methane and could be successively transferred with sustained methane production (**Figure 3B**). The rates of methane production (0.9 ± 0.6 μmol per day) by *G. hydrogenophilus* co-cultured with *M. barkeri* were lower, but comparable to rates previously observed (Rotaru et al., 2014b) in co-cultures of *G. metallireducens* and *M. barkeri* (2.7 ± 0.3 μmol per day).

**FIGURE 5:**
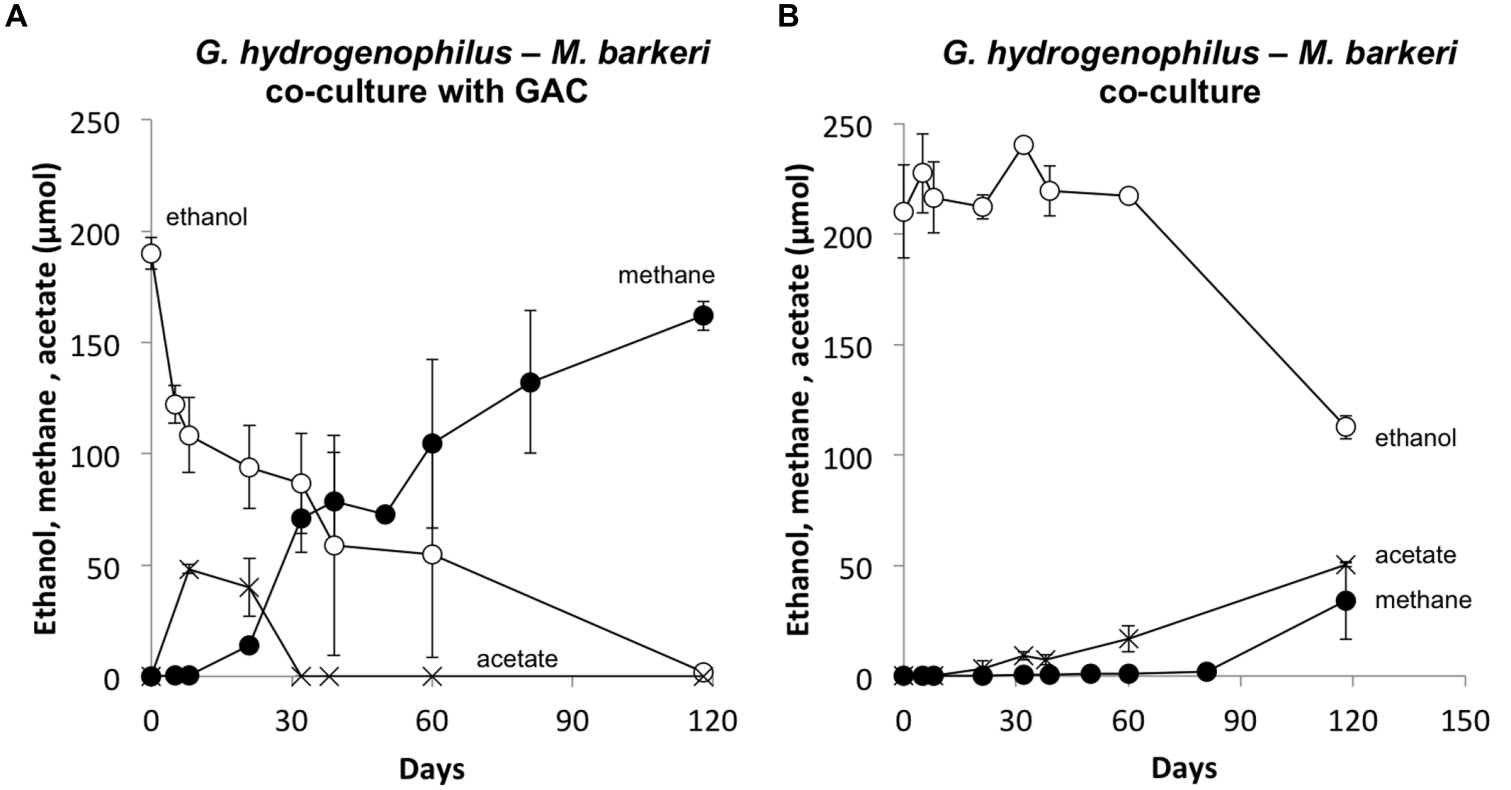
**Methane and acetate formation from ethanol in co-cultures of *G. hydrogenophilus* and *M. barkeri* with **(A)** or without **(B)** granular activated carbon.** Results are the mean and SD for triplicate incubations.

Several lines of evidence suggested that *G. hydrogenophilus* and *M. barkeri* exchanged electrons via DIET. For example, like *G. metallireducens* (Rotaru et al., 2014b), *G. hydrogenophilus* appeared to be incapable of exchanging electrons via H_2_ or formate because it did not form a successful co-culture with the strict H_2_/formate-utilizing methanogen *Methanospirillum hungatei*, even after 150 days of incubation (See Supplementary Materials). Furthermore, GAC greatly accelerated electron transfer between *G. hydrogenophilus* and *M. barkeri* (**Figure 5A**) compared to co-cultures initiated at the same time without GAC (**Figure 5**). The high conductivity of GAC promotes DIET (Liu et al., 2012; Rotaru et al., 2014b), but similar to other conductive materials, GAC is not expected to enhance interspecies H_2_ transfer (Chen et al., 2014a).

The availability of systems for genetic manipulation of *G. sulfurreducens* and *G. metallireducens* made if possible to further confirm electron transfer via DIET with deletions of genes for key extracellular electron transfer components (Summers et al., 2010; Rotaru et al., 2012, 2014a,b; Shrestha et al., 2013b). However, a strategy for genetic manipulation of *G. hydrogenophilus* has yet to be developed.

### Implications

The results demonstrate that *Geobacter* species differ substantially in their capacities for current production and Fe(III) oxide reduction, as well as their ability to form syntrophic associations via DIET. Among the species tested, the effectiveness for Fe(III) reduction was a poor predictor of their ability for current production. In contrast, the three species of *Geobacter* that produce the highest current densities are the only three *Geobacter* species among those tested to date that can participate in DIET.

The correspondence between the capacity for syntrophic growth and the ability to produce high current densities suggests that there are commonalities in these two types of extracellular electron exchange and that the prior evolution of some *Geobacter* species for syntrophic growth via DIET conferred characteristics that permit these species to effectively utilize electrodes as electron acceptors. Although electrically conductive pili are one component that is essential for high current densities and DIET (Malvankar and Lovley, 2014), it is likely that other extracellular electron transfer components, as well as features that favor cell aggregation/biofilm formation, are also important. Therefore, further elucidation of the mechanisms for DIET may also provide insights into how electrons are transferred through conductive electrode biofilms and vice versa.

## Conflict of Interest Statement

The authors declare that the research was conducted in the absence of any commercial or financial relationships that could be construed as a potential conflict of interest.
