# Supplementary material for: Link between capacity for current production and syntrophic growth in Geobacter species
Source: Front Microbiol. 2015 Jul 21;6:744. doi: 10.3389/fmicb.2015.00744 (PMC4523033; doi:10.3389/fmicb.2015.00744)
Supplement: Supplementary file 1 [file Data_Sheet_1.PDF]

Supplementary Material

Link Between Capacity for Current Production and Syntrophic Growth in *Geobacter* species

Amelia-Elena Rotaru[1,2]*, Trevor Woodard[1], Kelly P. Nevin[1], Derek R. Lovley[1]

[1]Department of Microbiology, University of Massachusetts, Amherst, Massachusetts, USA
[2]Nordic Center for Earth Evolution, Department of Biology, University of Southern Denmark, Odense, Denmark

*** Correspondence:** Amelia-Elena Rotaru, Nordic Center for Earth Evolution, Department of Biology, University of Southern Denmark, Campussvej 55, Odense, 5230, Denmark.
arotaru@biology.sdu.dk

1. Supplementary Figures

Supplementary Figure 1. Methane evolution from ethanol in three co-cultures of *G. hydrogenophilus* and *M. barkeri* during the first (A) and the third transfer (B). The black profiles show average of three incubations. The small white data points are measurements specific to each co-culture replicate.

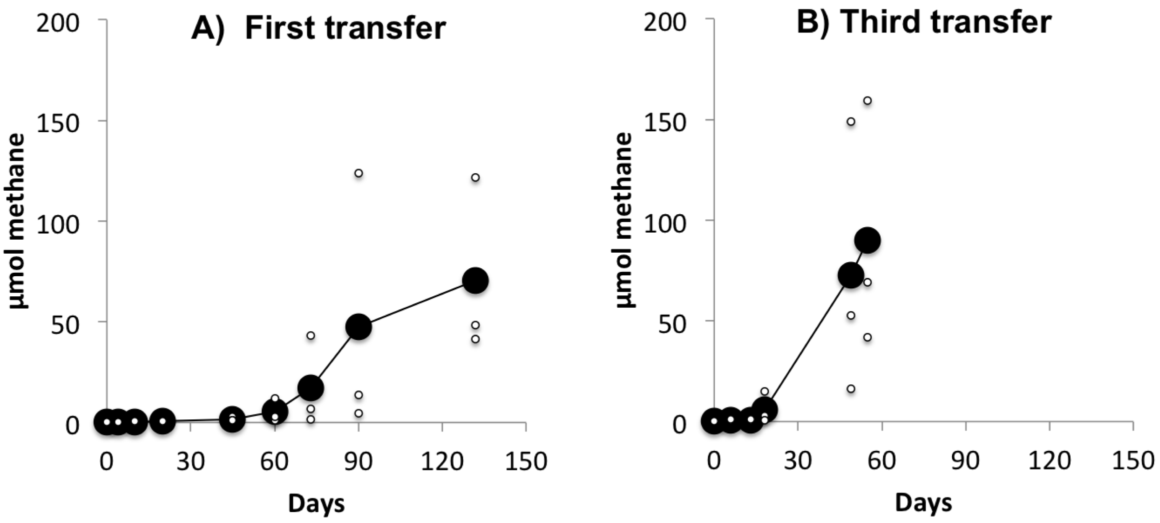

Fig. 1 SM

Supplementary Figure 2. Acetate formation from ethanol in cultures of *G. hydrogenophilus* incubated for 120 days with GAC (a) and *G. hydrogenophilus* incubated without GAC (b). Values are the result of triplicate incubations.

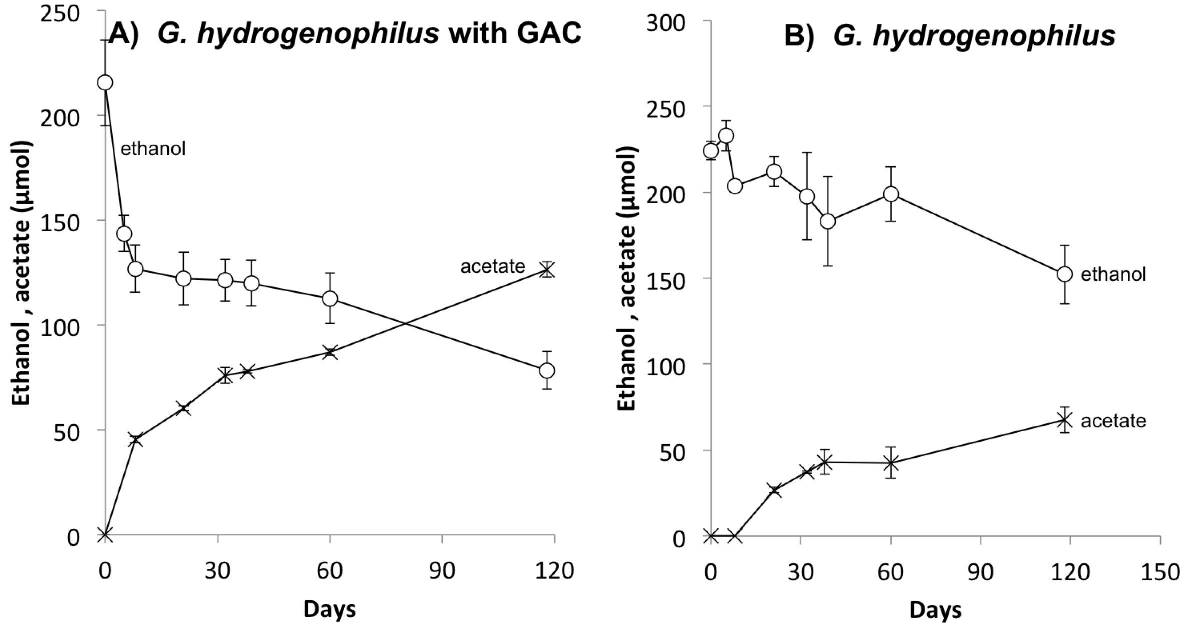

Fig. 2SM

Supplementary Figure 3. Absence of methane evolution in co-cultures of *Geobacter hydrogenophilus* and *Methanospirillum hungatei*.

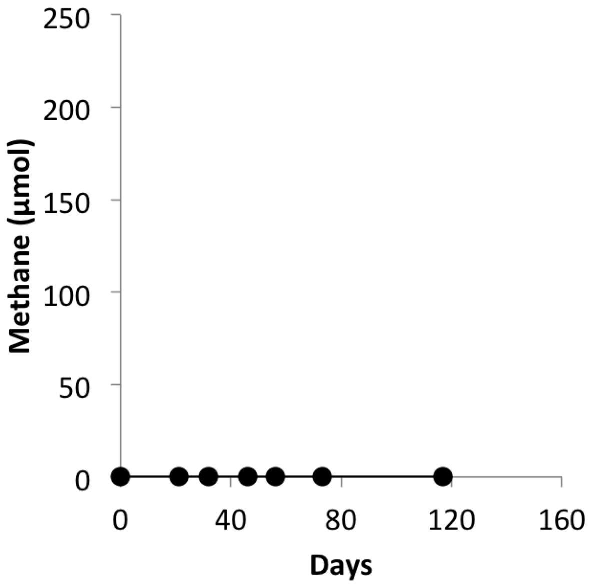

Fig. 3SM

frontiers
